# Editorial: Non-invasive Brain Stimulation and Plasticity Changes in Aging

**DOI:** 10.3389/fnagi.2016.00096

**Published:** 2016-05-04

**Authors:** David Bartrés-Faz, Simone Rossi, Alvaro Pascual-Leone

**Affiliations:** ^1^Unitat de Psicologia Médica, Departament de Medicina, Facultat de Medicina i Ciéncies de la Salut, Universitat de BarcelonaBarcelona, Spain; ^2^Unit of Neurology and Clinical Neurophysiology, Brain Investigation and Neuromodulation Lab. (Si-BIN Lab), Department of Neuroscience, University of SienaSiena, Italy; ^3^Berenson-Allen Center for Noninvasive Brain Stimulation and Division of Cognitive Neurology, Beth Israel Deaconess Medical Center, Harvard Medical SchoolBoston, MA, USA

**Keywords:** non-invasive brain stimulation, plasticity, cognition, life style, cognitive training, brain imaging

Our conceptualization of brain changes across the lifespan is evolving (Pascual-Leone et al., 2011). There appears to be no period when the brain and its functions are static. Instead, changes are continuous throughout the lifespan, some resulting in benefits, others in functional loss and decline (Park and Reuter-Lorenz, 2009; Pascual-Leone and Taylor, 2011). Therefore, the most suitable framework appears to be that of life-long, continued “developmental” processes that influence each other, and there is a growing need for deeper understanding of brain changes (plasticity) from prenatal states and infancy through childhood into adult and old age.

In humans, non-invasive brain stimulation (NIBS) techniques like transcranial magnetic stimulation (TMS) and transcranial direct current stimulation (tDCS) are well-suited to explore brain plasticity across the lifespan (Bashir et al.). First, they can provide valuable translatable physiologic biomarkers of the state of cortical reactivity, brain network connectivity and dynamics, and mechanisms of brain plasticity (Diester et al., 2015). Further, used alone or in combination with other approaches, like cognitive training, they can induce or modulate neuroplastic responses, which can reveal causal relations between brain and behavior, translate into behavioral modification, and even lead to new therapeutic interventions (Bartrés-Faz and Vidal-Piñeiro, 2016). The present research topic offers novel insights into this growing field of NIBS in the study of aging.

A first set of studies focus on motor cortex plasticity. Young-Bernier et al. hypothesize that measures of short-latency afferent inhibition (SAI), reflecting cortical cholinergic activity, mediate age-related individual differences to the plasticity-inducing effects of theta burst stimulation (TBS). While their results confirm high inter-individual variability to TBS, no association could be observed with responsiveness to SAI. Particular sample characteristics or unexplored specific responses to the stimulation protocols on aged individuals could account for these results, and further research is needed. Puri et al. investigated the role of the Val66Met BDNF polymorphism (encoding for a synaptic potentiation related neurotrophine) in motor cortical plasticity following tDCS. After 20, but not 10 min of stimulation, they observed greater motor plasticity responses to tDCS in the Met allele carriers, along with marked reduction in inter-individual variability. Hence, BDNF may modulate the time-course of motor plastic responses in elders and may be important to stratify sample selection where more homogeneous responses to NIBS are required (Shpektor et al.). Finally, Dickins et al. showed that the intermanual transfer of training simple motor skills is preserved in elders. However, for complex motor tasks, cortical excitability of the untrained hemisphere did only correlate with performance of the untrained hand in young individuals.

Moving to the realm of cognitive function, Chan et al. stress the relevance of obesity in advanced age as a condition aggravating age-related cognitive decline. As this effect is mediated through brain regions thought to sustain a relevant plastic role, the authors emphasize the need to promote plasticity, such as engaging in physical exercise. In a related study Arenaza-Urquijo et al. propose a theoretical perspective on brain and cognitive reserve postulates: amongst healthy aged subjects, exposure to lifetime enriched environments would mainly act through “neuroprotection,” leading to preserved brain function and structure. However, when considering patients at various stages of disease, higher reserve might translate to “compensatory” plasticity, providing greater capacity to minimize the clinical impact of pathology.

The last set of studies aims to enhance cognitive function in aging through the use of non-invasive interventions. Li et al. studied the effect of multimodal training (cognitive, physical, and social), whereas Lampit et al. investigated the impact of computerized cognitive training. In both reports significant behavioral improvements were observed that were related to changes in brain connectivity. Moreover Lampit et al. showed that cognitive-training induced functional brain changes preceded structural modifications, providing new evidences about the dynamics of the neurobiological underpinnings subtending cognitive training responses in elders.

Three studies examined the efficacy of tDCS to enhance cognitive functions in elders. Sandrini et al. investigated the effects of NIBS on reactivation of consolidated memories (i.e., reconsolidation). Compared to sham stimulation, tDCS with the anode over the dorsolateral prefrontal cortex (DLPFC) decreased the forgetting rate tested after 48 h and 1 month of initial memory encoding. However, combining brain stimulation with reconsolidation did not lead to better long-term memory performance. Following this pioneer work, further investigation is needed to determine if specific particular adaptations of the experimental reconsolidation designs are needed in elders. Fertonani et al. also applied tDCS targeting the DLPFC to investigate the time-course of plasticity effects on picture-naming tasks. They observed that young individuals benefited from stimulation when anodal tDCS was applied both during (i.e., *online*) and after task performance (i.e., *offline* stimulation), whereas for elder individuals the benefit was only observed during *online* stimulation. These results revealing an age-dependent timing of stimulation response may be considered in further cognitive neurorehabilitation studies. Also, the work suggest novel insights about neuromodulatory approaches to age-related cognitive dysfunction, by showing that *online* stimulation, thought to be associated with changes in membrane potential, was more effective in elders than effects primarily linked to synaptic potentiation (*offline stimulation*).

Finally, Meinzer et al. used anodal tDCS to enhance activity of M1 and investigated the impact on a word-generation task. The authors observed better performance both during anodal left M1 stimulation as well as during dual anodal left M1 and cathodal right M1, as compared to sham. Further, functional imaging data reflected that brain activity in premotor linguistic regions was enhanced, without effects in motor cortices. The findings hence, suggest that tDCS may have been acting as facilitating switching costs of two tasks that are engaging partially overlapping networks. They further show that tDCS can improve linguistic functions in aging by targeting brain sites (e.g., M1) functionally linked to the language system, but avoiding the typical perilesional stimulation sites in stroke patients.

In conclusion, the studies gathered in this Research Topic highlight the suitability of NIBS approaches to investigate the mechanisms and the consequences of plasticity associated to the aging process. From a methodological point of view, the findings contribute to our understanding of how major characteristics of the aging brain interact with stimulation parameters, hence stressing the need to consider distinct approaches when NIBS is to be applied to aged- as compared to young-populations. From a fundamental perspective, these studies provide novel insights into inter-individual differences in neuroplasticity, their relevance to the aging process and to behavioral and cognitive well-being in elders. In this regard, NIBS might even offer a novel intervention to promote cognitive function across the lifespan.

## Author contributions

DBF wrote a first draft of the text. SR and APL reviewed it and provided corrections and conceptual inputs.

## Funding

DBF was partially by research grant from the Spanish Ministerio de Economía y Competitividad (MINECO) ref. PSI2012-38257. APL was partly supported by the Sidney R. Baer Jr. Foundation, the NIH (R01HD069776, R01NS073601, R21 NS082870, R21 MH099196, R21 NS085491, R21 HD07616), the Football Players Health Study at Harvard University, and Harvard Catalyst; The Harvard Clinical and Translational Science Center (NCRR and the NCATS NIH, UL1 RR025758).

### Conflict of interest statement

The authors declare that the research was conducted in the absence of any commercial or financial relationships that could be construed as a potential conflict of interest.
